# A resource for benchmarking the usefulness of protein structure models

**DOI:** 10.1186/1471-2105-13-188

**Published:** 2012-08-02

**Authors:** Daniel Carbajo, Anna Tramontano

**Affiliations:** 1 Department of Physics, Sapienza University of Rome, P.le A. Moro, 5, 00185, Rome, Italy; 2 Institut Pasteur Fondazione Cenci-Bolognetti, Rome, Italy; 3 Center for Life Nano Science @Sapienza, Istituto Italiano di Tecnologia, Sapienza University of Rome, P.le A. Moro 5, 00185, Rome, Italy

**Keywords:** Protein structure prediction, Decoy database, Comparative modeling, ModelDB

## Abstract

**Background:**

Increasingly, biologists and biochemists use computational tools to design experiments to probe the function of proteins and/or to engineer them for a variety of different purposes. The most effective strategies rely on the knowledge of the three-dimensional structure of the protein of interest. However it is often the case that an experimental structure is not available and that models of different quality are used instead. On the other hand, the relationship between the quality of a model and its appropriate use is not easy to derive in general, and so far it has been analyzed in detail only for specific application.

**Results:**

This paper describes a database and related software tools that allow testing of a given structure based method on models of a protein representing different levels of accuracy. The comparison of the results of a computational experiment on the experimental structure and on a set of its decoy models will allow developers and users to assess which is the specific threshold of accuracy required to perform the task effectively.

**Conclusions:**

The ModelDB server automatically builds decoy models of different accuracy for a given protein of known structure and provides a set of useful tools for their analysis. Pre-computed data for a non-redundant set of deposited protein structures are available for analysis and download in the ModelDB database.

**Implementation, availability and requirements:**

Project name: A resource for benchmarking the usefulness of protein structure models. Project home page: http://bl210.caspur.it/MODEL-DB/MODEL-DB_web/MODindex.php.

Operating system(s): Platform independent. Programming language: Perl-BioPerl (program); mySQL, Perl DBI and DBD modules (database); php, JavaScript, Jmol scripting (web server). Other requirements: Java Runtime Environment v1.4 or later, Perl, BioPerl, CPAN modules, HHsearch, Modeller, LGA, NCBI Blast package, DSSP, Speedfill (Surfnet) and PSAIA. License: Free. Any restrictions to use by non-academics: No.

## Background

The function of a protein is brought about by its three-dimensional structure the knowledge of which can be instrumental to several applications, ranging from function assignment to the prediction of its mode of interaction with its molecular partners to the interpretation and design of re-engineering experiments.

The obvious limiting step in exploiting the power of protein structures in many of these applications clearly lies on the availability of a relatively small number of experimentally determined protein structures, stored in the PDB [1], compared to the number of known protein sequences [2]. This limitation can be partially overcome by the use of methods for inferring the structure of proteins from their sequences.

At present several methods are available to this end the most reliable of which remains comparative modeling, based on the observation that evolutionarily related proteins have similar structure and therefore that the knowledge of the structure of one member of a protein family (template) can be used as starting model for the others, provided that the evolutionary relationship can be detected at the sequence level. Because functional relevant regions are better preserved in evolution, the method has the advantage that it will produce better results for the biologically relevant parts of the target protein.

Comparative modeling has an additional advantage over other protein structure prediction methods: there is a known and well-studied relationship between the divergence between the sequences of two homologous proteins, indicative of their evolutionary distance, and the structural changes between the backbone atoms of their core [3]. This implies that, when a single template is used, it is possible to estimate beforehand the error affecting the model by measuring the percentage of identity between its sequence and that of the target protein.

The relationship has been validated several times, for example using the results of blind tests in the CASP (Critical Assessment of Methods of Protein Structure Prediction) series of initiatives [4]. The results of the experiments also repeatedly showed that the quality of models can be substantially improved when the whole set of sequences of the protein family are taken into account and this affects several steps of the modeling procedure, from the detection of the template to the quality of the alignment to the use of different regions from different templates in the final model. However, in this case and when multiple templates are used, the relationship between sequence and structure divergence is more difficult to estimate.

CASP has also tested the ability of independent methods to estimate the quality of models demonstrating that they can achieve a significant accuracy in selecting the best model among a set of diverse predictions for the same proteins, while methods for assigning an estimated quality to single models still lag behind [5].

It is obvious that the quality of a protein model dictates how effective it is for subsequent studies, however the identification of a precise and general relationship between the quality of a model and its usefulness for a specific application is still eluding the efforts of the community. The aim of the server described here is to allow developers of structure based methods to quickly test how well their method performs when models of different quality are used instead of experimental structures.

It has been shown that high-resolution models 1-2 Å RMSD away from the native counterpart can provide relevant functional information, such as the inference of enzyme reaction mechanisms [6] and the interpretation of disease-causing mutations [7]. In some cases they have been shown to be useful in ligand-docking studies [8], experimental structure determination aid [9,10] and drug design [11,12]. As accuracy drops, the range of applications narrows.

We reasoned that the easiest and most straightforward way to help solving the issue is to provide method developers with a curated and annotated set of models at different level of accuracy for each known protein structure to rapidly test the level of accuracy required for a model to be used in place of an experimental protein structure.

We describe here how we obtained models at different levels of quality for each of the proteins of known structure and introduce a tool, ModelDB, which allows easy access to them and to several relevant information about the modeled proteins.

The user can select entries on the basis of several annotations, structural and functional domains, gene ontology and Enzyme Commission Numbers extracted directly from publicly available databases.

The server also includes a tool to build a homology model of a protein of unknown structure and to compare the model with the template(s) used to build it.

## Methods

### Dataset

The initial dataset of proteins included proteins solved by X-ray crystallography alone or in complex with other molecules as available on January 3rd 2011, filtered not to contain any pair with more than 50% sequence identity (using PISCES [13]), excluding those structures with only Cα atoms, with a resolution worse than 2 Å, with a sequence length outside the range 20–10000 residues, and with an R-factor higher than 0.3. We were left with a total of 8,609 PDB chains. We could detect suitable templates, and therefore build comparative models, for 7,166 of them. Of these 2,999 have an EC number (72,648 in the whole PDB), 2,452 with a complete 4-digit EC number (63,474 in the whole PDB); 5,106 bind to ligands (97,388 in the whole PDB), 3,742 of them to more than one (70,780 in the whole PDB), and there are 1,199 different ligands found binding to this subset of modeled chains (9,891 in the whole PDB); 2,261 have at least one annotated catalytic site (51,437 in the whole PDB), 982 of them have more than one catalytic site (23,686 in the whole PDB); 4,546 are annotated in Swiss-Prot (125,966 in the whole PDB).

### Modeling strategy

The ModelDB modeling pipeline, written in Perl, relies on HHsearch [14] for template identification and Modeller [15-18] for building the complete model.

The sequence of each PDB chain is used as query in HHsearch to search for templates in the 70% non-redundant PDB database. All target-single template alignments with 80% minimum sequence coverage and 10^-1^ maximum E-value were used as input for Modeller to produce an all non-hydrogen atom single-template model for each of the selected sequences.

### Superposition of the models to the corresponding experimental structure

Each model of each target protein was compared to the corresponding experimental structure using LGA (Local–global Alignment) [19]. We record the GDT-TS and RMSD values and allow sorting the models according to these parameters (see later) as well as to HHsearch probability, E-value, score and coverage.

### Functional annotation

Each PDB structure in the input list (as well as its corresponding models) is annotated whenever possible at the residue level, using the CREDO database [20] that collects protein-ligand interactions, the Catalytic Site Atlas (CSA) [21,22] that includes information about enzyme catalytic sites, and Swiss-Prot.

### Model visualization

Experimental structures and their models can be visualized and colored at the residue level according to solvent accessibility and secondary structure as computed by DSSP [23], cavity occurrences and average depth as defined by Speedfill [24], and protrusion and burial indexes obtained via PSAIA [25].

This visualization is obtained using an in-house Perl program named mappON. A stand-alone version of mappON is also available to visualize the parameters described above and also the disorder probability (computed using DisEMBL [26]), evolutionary residue conservation and variability (retrieved from the ConSurf-DB [27]) on user provided structures. The tool is accessible via the ModelDB site.

Subsets of proteins can be selected on the basis of their functional and structural domains, GO annotation and Enzyme Commission Numbers.

### Model building

The user can build a homology model of a protein of unknown structure using Modeller [17] on the basis of templates identified using HHPred [14] and compare its structure with those of the templates used to build it taking advantage of all the described visualization tools.

## Results

### Database composition

Models for the 7,166 proteins obtained as described in Methods and annotated at the residue level with information about secondary structure, solvent accessibility, cavity occurrence, average depth and protrusion and burial indexes are stored in the ModelDB relational database.

**Figure 1:**
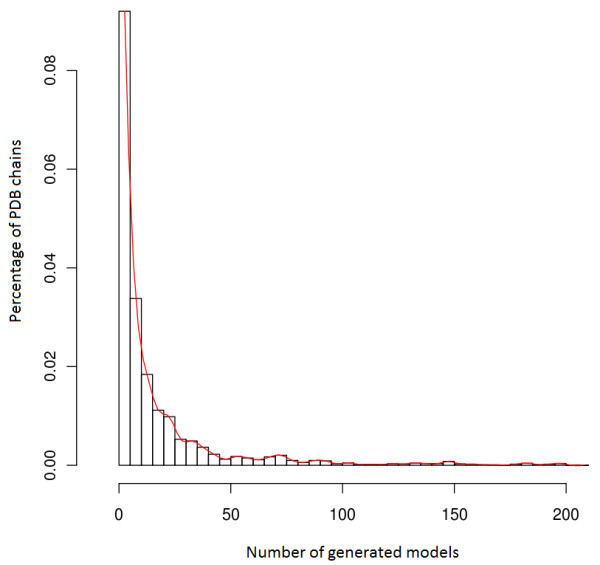
**Density distribution of the number of decoy models produced for the 7,166 PDB chains in the input list.** The majority of PDB chains have a number of decoy models between 0 and 10, the average being 17 and the maximum 206.

**Figure 2:**
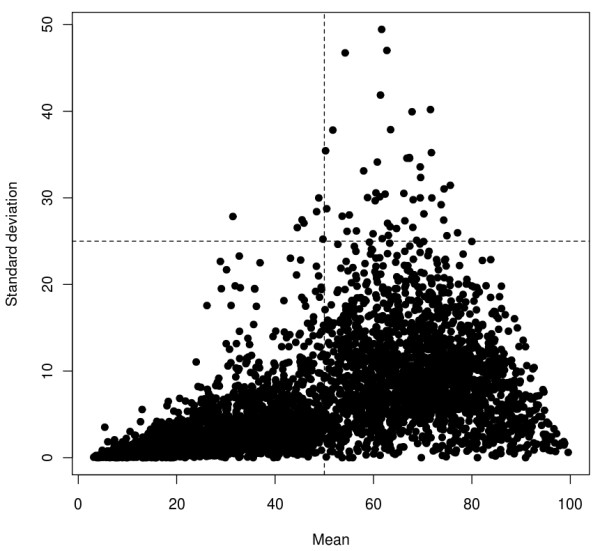
Scatter plot representing the mean vs. the standard deviation of GDT-TS in each decoy set.

The average number of models per PDB chain in the database is 17, the largest number of models being 206 for [PDB:2RHE] chain A. The distribution of the number of models is shown in Figure 1, while Figure 2 shows the distribution of the average GDT-TS and standard deviation values for the models in the database.

### ModelDB

The ModelDB database can be accessed via the publicly available ModelDB web server. Both can also be downloaded and installed locally.

#### ModelDB server

The ModelDB pipeline was used to build the pre-calculated model sets stored in the database and can be used to build models for user-provided structures. In this case, input sequences are subjected to the same procedure described in Methods for building the database.

The ModelDB web interface (http://bl210.caspur.it/MODEL-DB/MODEL-DB_web/MODindex.php) is conceived to be as user-friendly as possible and has several features. A user can either specify a PDB code or upload a protein structure of interest, in both cases the chain needs to be specified (by default the first chain present in the structure is analyzed).

If the input protein is not present in the database or the user changed the default parameters for modeling, the modeling program is launched. This is followed by a BLAST search with stringent parameters (90% coverage and an e-value of 10^-4^) against PDB and Swiss-Prot, to retrieve information and functional annotations for the protein entry or for a very close homologue.

Upon completion of these steps, the output page described next, which is directly displayed if the entry is already stored in the database, is shown.

**Figure 3:**
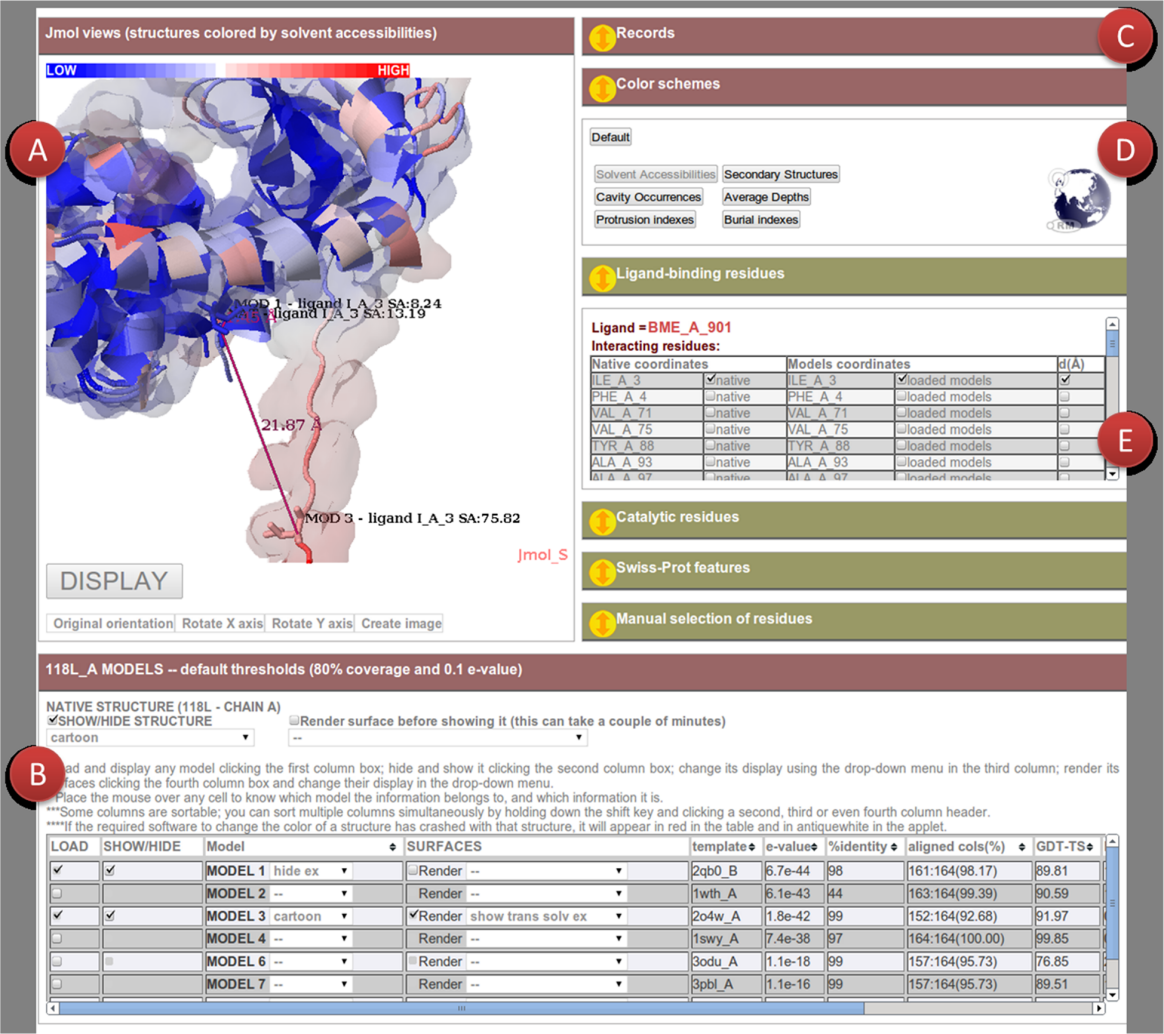
**ModelDB result page.** The structure of T4 lysozyme chain A (PDB code [PDB:118 l]) and of one of its decoy models (the third sorted according to HHsearch [14] score of the template used) are displayed in the Jmol window as cartoons and colored according to solvent accessibilities. For the model, the transparent solvent excluded surface is also shown. A ligand binding Isoleucine is highlighted in both structures, as well as in the most accurate model for the same protein. The Isoleucine in one of the models is predicted to be in an incorrectly modeled loop, far away from its correct position.

The page contains a short description of the protein and a sortable table (Figure 3B) where the models are listed and can be ranked. One or more models can be visualized using a Jmol applet (Figure 3A) and are shown superimposed to the experimental structure. Different representations are possible (cartoons, spacefill, trace, backbone representations, etc.) and solvent excluded and solvent accessible surfaces can be rendered. A state win-dow records what happens in the Jmol applet (Figure 3C). The user can also rotate the axes in the Jmol window and create images.

There is the possibility to color the structures and surfaces according to different coloring schemes (Figure 3D). Collapsible boxes provide functional annotation (Figure 3E). Functional residues as well as the distance in Å between corresponding Cαs of the experimental and modeled structure can be visualized in the structure(s). Finally, the models of a given protein can be downloaded as a zip file.

#### Some examples of application

We show here examples of how the ModelDB server can be used to identify the level of accuracy required for simple structure-based computations.

**Figure 4:**
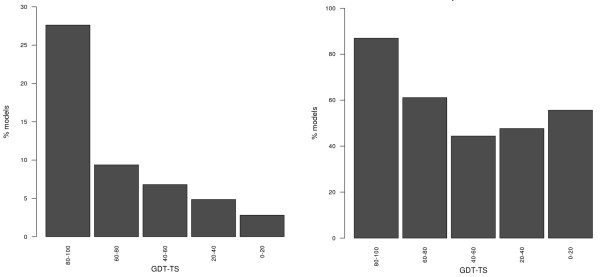
Percentage of protein models with different GDT-TS values in which at least 75% of the exposed (left) and buried (right) residues can be correctly identified.

For example, models are often used to identify suitable locations for modifications or functionalization of the protein. Therefore one could ask to which extent the classification between exposed and buried residues can still be made using a model and which is the minimum level of model quality required to obtain meaningful results. We defined exposed residues as those with a solvent accessibility value above 70% (and buried ones those with a value below 30%) with respect to the maximum residue value, as defined by Miller *et al*. [28]. As shown in Figure 4, one can correctly identify 75% of the exposed residues in more than 40% of models with a GDT-TS above 90, and in almost 30% of those with a GDT-TS above 80. Below the latter threshold, the percentage of models where at least 75% of the exposed residues are correctly detected reaches 10%. This is relevant to keep in mind when using models as frameworks for experiments.

**Figure 5:**
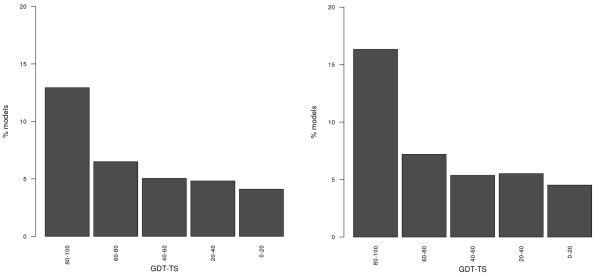
**Percentage of models with different GDT-TS values in which it is possible to correctly identify the largest surface cavity (at least 75% of its residues).** On the left results are shown for all entries in the database, while on the right only enzymes annotated in CSA [21,22] are considered.

Another common use of models concerns the identification of enzyme active sites. It is known that binding sites tend to occur in the largest cavity on the surface of proteins [29], so the obvious question is how well this property is conserved in models of different quality. Our data (Figure 5) show that only in a 20% of the models with a GDT-TS above 90 can at least 75% of the residues constituting the largest cavity be detected (a residue is considered to belong to a cavity if any of its atoms belongs to it). It follows that this approach is not very suitable for medium to low quality models and perhaps it should be parameterized differently for these cases. The situation moderately improves when the subset of enzymes stored in CSA is considered (Figure 5).

The last question we asked is whether the relative position of residues forming an active site, and therefore well conserved throughout evolution, can be reliably measured using models. This is relevant because in many cases the identification of specific residues at a given distance from each other are very good signs of the presence of an active site.

**Figure 6:**
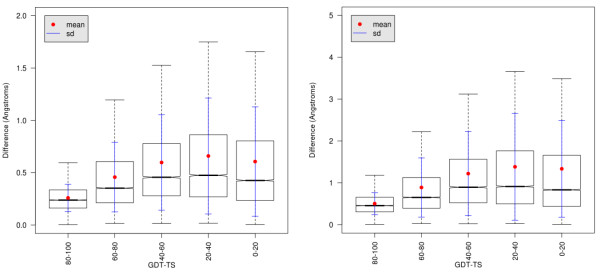
**Mean (left) and maximum (right) average Euclidean distance differences for residues of catalytic sites annotated in CSA**[21,22]**for models with different values of GDT-TS.**

We measured the Euclidean distance differences between every permutation of catalytic residue Cαs constituting an active site (with two or more residues) in the native structure and in its models of varying quality. Averaging all the differences over each active site showed an increasing mean Euclidean distance difference as model quality decreases in terms of GDT-TS (Figure 6). However, the maximum mean value of the difference per site (when model quality is the lowest) is never much higher than 0.5 Å, implying that the catalytic residues relative positions can be effectively estimated also in models of relatively low quality (Figure 6B).

## Conclusions

Since the gap between known protein sequences and structures continues to increase, researchers need to make use of protein structural models more routinely. Models usually contain structural inaccuracies that vary in number and severity, but they can still provide important insights into a protein role. There is no general rule that relates model accuracy with its usefulness for different applications, therefore there is the need to test the model quality tolerance for each specific structure-based method. ModelDB, the tool introduced here, serves this purpose by rapidly generating decoy sets for the proteins of interest. These decoys are intended to be used to test structure-based methods and decide to which extent each method can be applied to computed protein structure models. The tool allows the establishment of the quality threshold at which interpretable results, analogous to the ones that would be obtained with native structures, can be produced.

The project has involved the implementation of a pipeline divided in programs that work together, but also exist independently, either on-line or for local use when larger calculations are demanded. The ModelDB modeling pipeline takes a protein structure as input to generate single-template decoy models; it makes use of an in-house program named mappON to visualize the structures and the models colored according to different descriptors (solvent accessibilities, cavity occurrences, etc.). The on-line versions of both ModelDB and mappON query a relational database that not only contains pre-calculated decoy models, but also functional annotations extracted from different sources.

ModelDB contains decoy models created for a significant subset of the PDB, thereby covering a significant portion of the protein structural space compared to the other resources; this portion will increase as new decoy sets will be built and stored in the database. Individual decoy sets themselves are expected to cover wider quality ranges in new releases as more structures are deposited in the PDB. Last but not least, ModelDB also provides a visualization window where any decoy in a set, colored according to different descriptors, can be loaded, inspected and compared with its native counterpart.

## Competing interests

The authors declare that they have no competing interests.

## Authors’ contributions

AT designed the study. DC implemented the method. AT and DC wrote the paper. Both authors have read and approved the final manuscript.
